# Genetic diversity of *Plasmodium vivax* metacaspase 1 and *Plasmodium vivax* multi-drug resistance 1 genes of field isolates from Mauritania, Sudan and Oman

**DOI:** 10.1186/s12936-017-1687-1

**Published:** 2017-02-02

**Authors:** Fatimata Sow, Guillaume Bonnot, Bilal Rabah Ahmed, Sidi Mohamed Diagana, Hachim Kebe, Mohamedou Koita, Ba Malado Samba, Said K. Al-Mukhaini, Majed Al-Zadjali, Seif S. Al-Abri, Osama A. M. Ali, Abdallah M. Samy, Muzamil Mahdi Abdel Hamid, Musab M. Ali Albsheer, Bruno Simon, Anne-Lise Bienvenu, Eskild Petersen, Stéphane Picot

**Affiliations:** 1 Institut de Chimie et Biochimie Moléculaires et Supramoléculaires ICBMS-UMR5246, CNRS-INSA-CPE, Malaria Research Unit, University Claude Bernard Lyon 1, 43 Boulevard du 11 novembre 1918, Lyon, 69622 Villeurbane France; 2 Laboratoire de Bactériologie et Parasitologie de l’Hôpital Cheikh Zayed, BP-5720 Nouakchott, Mauritania; 3 Service des Maladies Infectieuses et Tropicales, Centre Hospitalier National de Nouakchott, BP-612 Nouakchott, Mauritania; 4 Laboratoire de Parasitologie et de Mycologie Médicale Institut National de Recherches en Santé Publique (INRSP), Avenue Jemal AbdeNasser, BP-695 Nouakchott, Mauritania; 5 Laboratoire Analyse de Biologie Médicale du Centre hospitalier de Rosso Mauritanie, BP-41 Rosso, Mauritania; 6 Department of Infectious Diseases, The Royal Hospital, Muscat, Oman; 7 Department of malaria, Ministry of Health, Muscat, Oman; 8 Entomology Department, Faculty of Science, Ain Shams University, Abbassia, Cairo, 11566 Egypt; 9 Biodiversity Institute, University of Kansas, Lawrence, KS 66045 USA; 10 Department of Parasitology and Medical Entomology, Institute of Endemic Diseases, Medical Campus, University of Khartoum, Qassr Street, P.O. BOX 102, Khartoum, Sudan; 11 Institute of Clinical Medicine, Faculty of Health Science, University of Aarhus, Aarhus, Denmark; 12 Institut of Parasitology and Medical Mycology, Hôpital de la Croix-Rousse, Hospices Civils de Lyon, Lyon, France

**Keywords:** *Plasmodium vivax*, Metacaspases, PvMCA1-cd, *pvmdr1* gene, SNP, Drug resistance, Apoptose, Oman, Mauritania, Sudan

## Abstract

**Background:**

*Plasmodium vivax* is the second most important human malaria parasite, widely spread across the world. This parasite is associated with important issues in the process toward malaria elimination, including potential for relapse and increased resistance to chloroquine. *Plasmodium vivax* multi-drug resistant (*pvmdr1*) is suspected to be a marker of resistance although definitive evidence is lacking. Progress has been made in knowledge of biological factors affecting parasite growth, including mechanisms of regulated cell death and the suspected role of metacaspase. *Plasmodium vivax* metacaspase1 (PvMCA1-cd) has been described with a catalytic domain composed of histidine (H372) and cysteine (C428) residues. The aim of this study was to test for a link between the conserved histidine and cysteine residues in PvMCA1-cd, and the polymorphism of the *P. vivax* multi-drug resistant gene (*pvmdr1*).

**Results:**

Thirty *P. vivax* isolates were collected from Mauritania, Sudan, and Oman. Among the 28 *P. vivax* isolates successfully sequenced, only 4 samples showed the conserved His (372)–Cys (428) residues in PvMCA1-cd. Single nucleotide polymorphisms observed were H372**T** (46.4%), H372**D** (39.3%), and C428**R** (85.7%). A new polymorphic catalytic domain was observed at His (282)–Cys (305) residues. Sequences alignment analysis of *pvmdr1* showed SNP in the three codons 958, 976 and 1076. A single SNP was identified at the codon M958Y (60%), 2 SNPs were found at the position 976: Y976**F** (13%) and Y976**V** (57%), and 3 SNPs were identified at the position 1076: F1076**L** (40%), F1076**T** (53%) and F1076**I** (3%). Only one isolate was wildtype in all three codons (MYF), 27% were single MY**L** mutants, and 10% were double M**FL** mutants. Three new haplotypes were also identified: the triple mutant **YVT** was most prevalent (53.3%) distributed in the three countries, while triple **YFL** and **YVI** mutants (3%), were only found in samples from Sudan and Mauritania.

**Conclusions:**

Triple or quadruple mutants for metacaspase genes and double or triple mutants for *Pvmdr1* were observed in 24/28 and 19/28 samples. There was no difference in the frequency of mutations between PvMCA1-cd and *Pvmdr1* (P > 0.2). Histidine and cysteine residues in PvMCA1-cd are highly polymorphic and linkage disequilibrium with SNPs of *Pvmdr1* gene may be expected from these three areas with different patterns of *P. vivax* transmission.

## Background

*Plasmodium vivax* has the broadest geographic distribution worldwide, and is a rising issue outside sub-Saharan Africa [1]. According to the World Health Organization (WHO), more than a third of the world’s population, mostly in Asia and Latin America, is at risk of *P. vivax* malaria infection. In 2013, this parasite was responsible for more than one million cases in four countries (Ethiopia, India, Indonesia and Pakistan) [2, 3]. Several years ago, *P. vivax* was thought to be absent in sub-Saharan Africa based on the fact that this parasite uses the human Duffy antigen/chemokine receptor (DARC) to invade red blood cells and most African people rarely express this receptor. However in the Saharan area, Berber peoples (Moors, Tuareg, mostly Duffy-positive blood groups) were found infected by *P. vivax* [4, 5]. The presence of *P. vivax* malaria in Mauritania was reported first in 1948 [6], and recent studies have shown the predominance of *P. vivax* malaria in Nouakchott, Mauritania [7–10].

In Eastern Africa, cases of *P. vivax* malaria are mostly reported from Ethiopia and Eritrea, reports of *P. vivax* malaria in Sudan are few [11]. However, one of the characteristics of *P. vivax* infection is the low parasite densities, leading researchers to underestimate the real prevalence of *P. vivax* infection in endemic areas. The Sultanate of Oman has long been an area of vivax malaria transmission (33,000 cases in 1990), but was declared free from malaria transmission in 2004. However, a focus of local transmission was found in September 2007 and secondary cases occurred in 2010, in 2011 and in 2012. The local cases most probably reflect the high number of migrant workers from the Indian subcontinent living and visiting their home countries where malaria especially *P. vivax* is endemic. Recently, the Disease Surveillance and Control service at the Ministry of Health at Muscat was informed of vivax malaria cases in Maabaila in Seeb area, Muscat.

Chloroquine and primaquine have been used to treat *P. vivax* malaria infection for both asexual and liver stages [5, 12]. The first reports on chloroquine resistance in *P. vivax* were from Papua New Guinea (PNG) and Indonesia in 1989 and 1991, respectively. Subsequently, cases of chloroquine resistance have been reported in several areas [13–15].

No report yet exists of *P. vivax* chloroquine resistance in Sudan and Mauritania [12, 16], but *pvmdr1* mutations are emerging according to observations of imported cases from these countries. Several studies have reported the link between drug resistance and polymorphisms in *pvmdr1* gene at codons Y976F and F1076L. *Pvmdr1* mutated codon 976F was linked to treatment failure in studies conducted in Southeast Asia and Ethiopia [17, 18]. However, still no consensus exists regarding the relationship between polymorphism at codons 976 and 1076 in the *pvmdr1* gene and drug resistance or clinical failures [19, 20].

Considering the emergence of drug resistance of *P. vivax* and the gaps of knowledge about its biology, studies of mechanisms of parasite death to seek new drug targets are much needed. About approximately two decades ago, the first evidence of *Plasmodium falciparum* parasite apoptosis by DNA fragmentation after drug pressure was reported [21]. Only chloroquine sensitive clones were reported to undergo apoptosis, while chloroquine resistant clones died by necrosis. These findings opened the door to a potential relationship between *Plasmodium* resistance and apoptosis, suggesting that resistance could be associated with default of apoptosis. Apoptosis is a regulated cell death, that is central to the development and homeostasis in metazoans [22]. Apoptosis was thought to be limited to multicellular organisms, but several studies have now provided evidence that apoptosis occurs in many unicellular organisms [21, 23–27].

Caspases are a family of proteases of cysteine that plays a key role in the execution of apoptosis [28]. A caspase-like family has been identified in plant, fungi and protozoa, and has been named “metacaspases” [29]. *In silico* studies have shown that metacaspases are structurally close to caspases and classified in C14 family, clan CD. The C14 family is characterized by histidine and cysteine residues in the catalytic dyad [29–31].

In the genome of *P. falciparum*, three metacaspases (MCA) were identified (PfMCA1-3), but only PfMCA1 was shown to possess histidine and cysteine residues required for the catalytic activity and to be involved in apoptosis of *P. falciparum* [32]. Likewise, in the *P. vivax* genome, three metacaspases have also been described and named PvMC1 (Pv114725), PvMC2 (Pv118575) and PvMC3 (Pv08564), respectively. Multiple alignment of the predicted caspase domains of the two human malaria parasites, *P. falciparum* (PfMCA1-3) and *P. vivax* (PvMCA1-3) and the murine malaria parasite *Plasmodium berghei* (PbMCA1-3) has shown that only PfMCA1, PvMCA1 and PbMCA1 possess the dyad histidine–cysteine conserved in the catalytic domain [33]. The proteolytic activity of the catalytic domain of PfMCA1 was studied in yca1 deficient *Saccharomyces cerevisiae,* and led to growth retardation and a drastic yeast cell death [34]. However, little is known about the involvement of PvMCA1 expression in the life and death of the *P. vivax* parasite.

Sequence alignment of PVX-114725 (Salvador 1 *P. vivax* metacaspase) with PF13-0289 (*P*. *falciparum* metacaspase 1) and isolates of *P. vivax* caspase-like from Mauritania (PVMG-03834), Brazil (PVBG-03488), India (PVIIG-01002) and North Korea (PVNG-00719) has shown a high similarity in the catalytic domain and a conserved histidine and cysteine residues. The catalytic histidine/cysteine dyad of PfMCA1 is found at positions 404 and 460 in the coding sequence, while in the PvMCA1-cd of *P. vivax* Sal 1 and *P. vivax* caspase like-isolates from the four endemic countries, these residues are found at positions 372 and 428. Interestingly, a new putative histidine/cysteine dyad was identified at positions 282 and 305. The objective of the present study was to investigate the relationship between the polymorphism found in histidine and cysteine residues of the catalytic domain of *P. vivax* (PvMCA1-cd) with the putative marker of drug resistance *pvmdr1* gene in samples collected from three geographically distant countries (Mauritania, Sudan and Oman).

## Methods

### Database accession of strains and isolates used in this study

Metacaspase sequences typical of distinct endemic areas were collected from Genbank: *P. vivax* metacaspase1 Salvador 1 (Sal-1, Central America, accession PlasmoDB: PVX_114725); *P. vivax* caspase-like Mauritania I (West Africa, accession UniprotKB: PVMG-03834), *P. vivax* caspase-like Brazil I (South America, accession UniprotKB: PVBG-03488); *P. vivax* caspase-like India VII (Asia, accession UniprotKB: PVIIG-01002) and *P. vivax* caspase-like North Korean (Asia, accession UniprotKB: PVNG-00719), and used as references in alignment. *P. vivax* multidrug-resistant gene 1: *pvmdr1* (accession: GenBank: AY618622) was used for resistance marker analysis. *Plasmodium falciparum* metacaspase1 isolated from 3D7 clone (accession: PlasmoDB: PF13-0289) was used for comparison.

### Blood sample collection

**Fig. 1:**
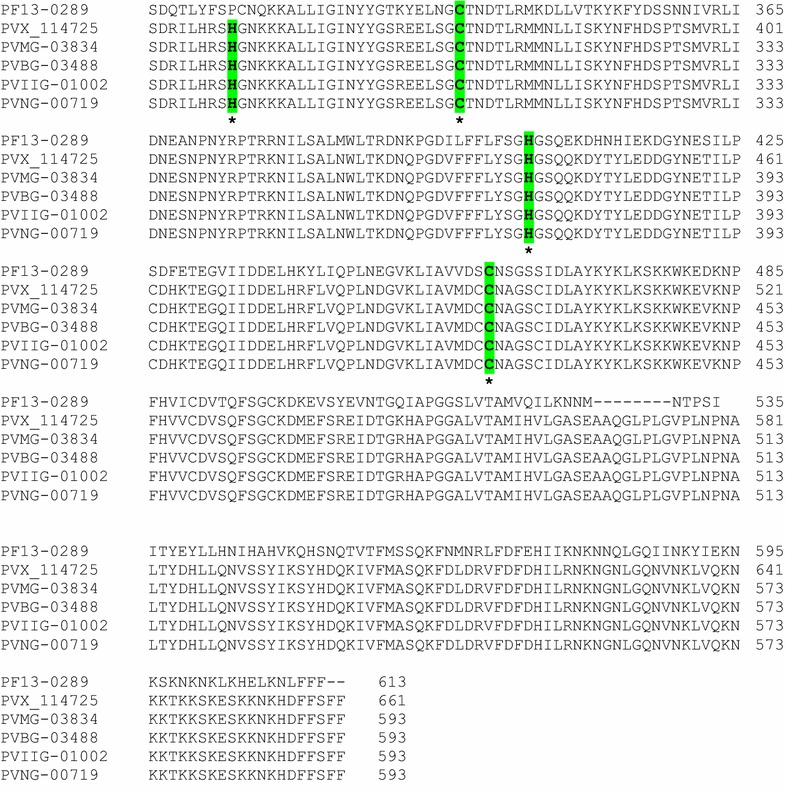
Alignment histidine and cysteine residues in the catalytic domain of metacaspases. The universally conserved histidine (His) and cysteine (Cys) residues are marked by a star in the catalytic site. In the PfMCA1 catalytic site, His–Cys are found at position 404 and 460. In the PvMCA1-cd, His–Cys, were identified at position 372 and 428, as well as in *P. vivax* from Mauritania, Brazil, India and North Korea. A second potential putative catalytic site His–Cys was also identified upstream of the catalytic domain at positions 282-305 in the five *P. vivax* sequences. Accessions of reference strains are PF13-0289 (ABC84559), PVX-114725 (EDL46606), PVMG-03834 (KMZ92479), PVBG-03488 (KMZ86023), PVIIG-01002 (KMZ79728), PVNG-00719 (KMZ98925)

Samples were collected from three malaria endemic or non-endemic countries: Mauritania, Sudan and Oman (Fig. 1). In Mauritania, the study was conducted in the summer period (July to September 2015) in three health centers located in Nouakchott: Hôpital Cheikh Zayed, Centre Santé de Teyarett and Hôpital Mère-Enfant. After informed consent was obtained from all patients and/or guardians of children, questionnaires were used to record patient information (age, sex, body temperature, fever, histories of the illness and medical examination). Blood samples were collected by finger prick and were spotted on Whatman^®^ filter papers, and dried and stored until use. Thick and thin smears were prepared and read by microscopy of Giemsa-stained blood films for malaria diagnostic and species of *Plasmodium* were recorded. Among the 20 *P. vivax* isolates collected during this study, 10 were chosen according to their highest parasite density. Samples from Sudan were provided by the Institute of Endemic Diseases, University of Khartoum, Khartoum, Sudan. These samples were collected from malaria patients in Whatman filter papers during 2013–2014. Samples from Oman were obtained from slides of Giemsa-stained thick blood film from patients of the Indian-subcontinental origin (India, Pakistan and Bangladesh) collected in Maabaila in the Seeb area, in Muscat, during the same period.

### DNA preparation from blood samples

DNA from Mauritania and Sudan samples was extracted from blood-spot samples on filter paper with Instagene Matrix Resin (Bio-Rad, France), according to the manufacturer’s instructions. The identification of species was confirmed by real-time PCR using species-specific primers [35]. DNA from thick blood films (Oman) was extracted as described previously in the literature [36].

### PCR amplification of PvMCA1 gene and *Pvmdr1* gene

Real-time polymerase chain reaction (PCR) was performed using SYBR Green I dye binding specifically to double-stranded DNA. Two pairs primers were used to amplify PvMCA1-cd and *pvmdr1* Primers were designed and synthesized by TIB Molbiol (France). Primers sequences and PCR program are presented in Table 1.

**Table 1:** Primers used for the amplification of PvMCA1-cd, *pvmdr1,* and *Pvcsp* of *P. vivax* isolates

Genes	Primers	Sequences	PCR program
PvMCA1-cd	MCA1-F1	5′-CATGGAAACAAAAAAAAGG-3′	95 °C 10′, 35 cycles 94 °C 30″ 56 °C 30″ 72 °C 2′
	MCA1-R1	5′-CGAAAACTCCATATCTTTGC-3′	
*pvmdr1*	mdr1-F1	5′-ATAGTCATGCCCCAGGATTG-3′	95 °C 10′, 45 cycles 95 °C 10″, 60 °C 10″ and 72 °C 30″
	mdr1-R1	5′-ACGTTTGGTCTGGACAAGTAT-3′	
*Pvcsp*	VCSP-OF	5′-ATGTAGATCTGTCCAAGGCCATAAA-3′	Primary reaction
	VCSP-OR	5′-TAATTGAATAATGCTAGGACTAACAATATG-3′	95 °C 10′, 40 cycles 95 °C 1′, 58 °C 2′ and 72 °C 2′
	VCSP-NF	5′-GCAGAACCAAAAAATCCACGTGAAAATAAG-3′	Nested reaction
	VCSP-NR	5′-CCAACGGTAGCTCTAACTTTATCTAGGTAT-3′	30 cycles 95 °C 1′, 62 °C 2′ and 72 °C 2′

*PvMCA1*-cd: catalytic domain of Metacaspase1’s *P. vivax*

*Pvmdr1*: multidrug-resistant of *P. vivax*

Pvcsp: circumsporozoite surface protein gene of *P. vivax*

’: minute

”: second

°C: degree Celsius

Real-time polymerase chain reactions were performed in a total volume of 20 µl containing 1 µmol/l of each primer, 3 mmol/l MgCl_2_, and 2 µl of Light Cycler Fast Start DNA Master SYBR Green I buffer (Roche Molecular Biochemicals). Five microliters of genomic DNA were added to the PCR mixtures. Before sequencing, all PCR products were separated on agarose gel (1.5%), and expected sizes of PvMCA1-cd and *pvmdr1* genes were purified using Extraction Kit (Qiagen, France). Sequencing of PvMCA1-cd and *Pvmdr1* genes was carried out by the Biofidal Company (Biofidal, France).

### *Pvcsp* genotyping of *P. vivax* isolates

Extracted DNA was first submitted to PCR for *Plasmodium* genus and *Plasmodium* species detection, as described previously [35]. Oligonucleotide primers and PCR conditions are described in Table 1.

All amplification reactions were carried out in a total volume of 20 µl and the presence of 250 nM of each oligonucleotide primers for *Pvcsp* and 2.0 µl of Light Cycler Fast Start DNA Master SYBR Green 1 reaction mix. Primary amplification reactions were initiated with 5.0 µl of the template genomic DNA, and 1.0 µl of the product of these reactions was used to initiate the secondary amplification reactions. The cycling parameters for PCR were as follows: an initial denaturation step at 95 °C for 10 min preceded the cycles of annealing at a temperature defined for each primer pair (Table 1) for 2 min, extension step at 72 °C for 2 min, and a denaturation step at 95 °C for 1 min. After a final annealing step followed by 5 min of extension, reaction mixtures from each capillary were collected and stored at 4 °C until secondary PCR or sequencing analysis.

The sequences were determined directly from the PCR-purified templates using Qiagen DNA purification kit, according to the manufacturer’s instructions. Direct sequencing of the full length of *pvcsp* was performed in both directions using a 3730 XL DNA analyzer (Applied Biosystems). Sequences were confirmed by BLAST and nucleotide sequences were translated into amino acid sequences. BioEdit version 7.2.5 was used to analyze and control the DNA sequences. MUSCLE (MUltiple Sequence Comparison by Log-Expectation) was used for sequence alignment. MEGA 7, version 7.0.14, was used for phylogenetic analysis. Phylogenetic tree was performed using Maximum Likelihood method implemented in MEGA7.

## Results

### Analysis of *pvmdr1* gene polymorphisms

The *pvmdr1* gene was successfully sequenced in e 30 *P. vivax* isolates. Sequence analysis of *pvmdr1* showed SNPs at codons 958, 976 and 1076 (Table 2 *)*. Haplotypes with a single mutation were identified at the codon position M958**Y** (60%), haplotypes with two mutations were found at the position 976: Y976**F** (13%) and Y976 **V** (57%) and three SNPs were identified at the position 1076: F1076**L** (40%), F1076**T** (53%) and F1076**I** (3%). Y976**F** and F1076**I** mutants were not identified in isolates from Oman. Only one isolate showing a wild-type at all three codons (**MYF**) was from Mauritania.

**Table 2:** Single nucleotide polymorphisms identified in the *pvmdr1* gene

Location/ID-isolate	M958Y	Y976F/Y976V	F1076L/F1076I/F1076T
NCBI-AY618622^a^	M	Y	F
MAURIT-61606	M	Y	F
MAURIT-00721	M	Y	**L**
SUDAN-KH-179	M	Y	**L**
SUDAN-KH-193	M	Y	**L**
OMAN-8	M	Y	**L**
OMAN-39	M	Y	**L**
OMAN-3	M	Y	**L**
OMAN-51	M	Y	**L**
OMAN-48	M	Y	**L**
SUDAN-KH-144	M	**F**	**L**
SUDAN-KH-142	M	**F**	**L**
SUDAN-KH047	M	**F**	**L**
SUDAN-KH-145	**Y**	**F**	**L**
MAURIT-06727	**Y**	**V**	**I**
MAURIT-Mou15	**Y**	**V**	**T**
MAURIT-06826	**Y**	**V**	**T**
MAURIT-Chi43	**Y**	**V**	**T**
MAURIT-Well30	**Y**	**V**	**T**
MAURIT-94241	**Y**	**V**	**T**
MAURIT-Jid34	**Y**	**V**	**T**
MAURIT-63933	**Y**	**V**	**T**
SUDAN-KH-130	**Y**	**V**	**T**
SUDAN-KH-173	**Y**	**V**	**T**
SUDAN-KH-103	**Y**	**V**	**T**
SUDAN-KH-195	**Y**	**V**	**T**
OMAN-52	**Y**	**V**	**T**
OMAN-36	**Y**	**V**	**T**
OMAN-13	**Y**	**V**	**T**
OMAN-17	**Y**	**V**	**T**
OMAN-47	**Y**	**V**	**T**

Residues that differ from the wild-type *P. vivax* Sal-I strain are indicated in bold type

Nonsynonymous substitutions: **Y** (Tyrosine), **F** (Phenylalanine), **V** (Valine), **L** (Leucine), **I** (Isoleucine), **T** (Threonine)

*MAURIT* Mauritania, *ID* identification

^a^GenBank accession no. AY618622 (wild-type strain)

The haplotypes **YVT** (triple mutant) was the most frequent (16/30, 53.3%), followed by **MYL** (single mutant) (8/30, 26.6%) and both were observed in the three countries. The **MFL** haplotypes (double mutant) were observed only in Sudan (3/30, 10%). **YFL** and **YVI** were only found once in Sudan and in Mauritania, respectively.

### Comparison of *P. vivax* caspase-like sequences of reference strains from different areas

Sequence alignment of *P. vivax* Sal-1 metacaspase 1 with caspase-like sequences of *P. vivax* isolates from Mauritania I (PVMG-03834), Brazil I (PBG-03488), India (PVIIG-01002), North Korea (PVNG-00719) and *P. falciparum* metacaspase 1 (PF13-0289) has shown the universally conserved histidine and cysteine residues in the catalytic dyad at the position 372 and 428. Interestingly, this analysis revealed a new second putative site of histidine/cysteine catalytic dyad at the position 282 and 305 (Fig. 1).

### Analysis of PvMCA1 catalytic domain

The catalytic domain of metacaspase 1 gene (PvMCA1-cd) was successfully sequenced in 28 *P. vivax* isolates. Sequences analysis of PvMCA1-cd showed SNPs in the catalytic domain (Table 3). Among the 28 *P. vivax* isolates, only four (one isolate from Mauritania and Oman and two from Sudan) showed conserved His (372)–Cys (428) residues in the catalytic domain and in the new putative site His (282)–Cys (305) (4/28, 14%). The two SNPs found in the H372 residue were H372**T** (13/28, 46.4%) and H372**D** (11/28, 39.3%). The only single SNP at position C428 was C428**R** (24/28, 85.7%).

**Table 3:** Single nucleotide polymorphisms found in PvMCA1-cd gene in comparison with *pvmdr1* gene

Location/ID-isolate	H282M/H282K	C305T/C305R	H372D/H372T	C428R	*pvmdr1*
PlasmoDB-PVX_114725^a^	H	C	H	C	MYF
MAURIT-00721	H	C	H	C	MY**L**
SUDAN-KH-142	H	C	H	C	M**FL**
SUDAN-KH-193	H	C	H	C	MY**L**
OMAN-48	H	C	H	C	MY**L**
MAURIT-06727	H	**T**	**D**	**R**	**YVI**
MAURIT-Jid34	H	**T**	**D**	**R**	**YVT**
MAURIT-63933	H	**T**	**D**	**R**	**YVT**
SUDAN-KH-195	H	**T**	**D**	**R**	**YVT**
OMAN-36	H	**T**	**D**	**R**	**YVT**
OMAN-13	H	**T**	**D**	**R**	**YVT**
OMAN-17	H	**T**	**D**	**R**	**YVT**
OMAN-51	ND	**T**	**D**	**R**	MY**L**
SUDAN-KH-103	ND	**T**	**D**	**R**	**YVT**
MAURIT-94241	**M**	**T**	**D**	**R**	**YVT**
MAURIT-Well30	**M**	**T**	**D**	**R**	**YVT**
MAURIT-Mou15	**K**	**R**	**T**	**R**	**YVT**
MAURIT-06826	**K**	**R**	**T**	**R**	**YVT**
MAURIT-61606	**K**	**R**	**T**	**R**	MYF
MAURIT-Chi43	**K**	**R**	**T**	**R**	**YVT**
SUDAN-KH-179	**K**	**R**	**T**	**R**	MY**L**
SUDAN-KH-144	**K**	**R**	**T**	**R**	M**FL**
SUDAN-KH-145	**K**	**R**	**T**	**R**	**YFL**
SUDAN-KH-130	**K**	**R**	**T**	**R**	**YVT**
SUDAN-KH-173	**K**	**R**	**T**	**R**	**YVT**
OMAN-52	**K**	**R**	**T**	**R**	**YVT**
OMAN-8	**K**	**R**	**T**	**R**	MY**L**
OMAN-39	**K**	**R**	**T**	**R**	MY**L**
OMAN-3	**K**	**R**	**T**	**R**	MY**L**

Residues that differ from wild-type *P. vivax* Sal-1 strain are indicated in bold type

Nonsynonymous substitutions: **K** (Lysine), **M** (Methionine), **R** (Arginine), **T** (Threonine), **D** (Acide aspartique)

*MAURIT* Mauritania, *ID* identification, *ND* no-determined

^a^PVX_114725: PlasmoDB annotation (wild-type strain Sal-1)

At the second putative catalytic dyad, the SNPs identified at H282 and C305 residues were H282**K** (50%), H282**M** (8%) and C305**R** (50%), C305**T** (42%)

Therefore, the distribution of these mutations in the three different countries can be shown as following: at the 372–428 catalytic site, the TR double mutant was predominant (13/28; 31% from Mauritania and Oman and 38% from Sudan), followed by DR (11/28; 45% from Mauritania, 36% from Oman, 18% from Sudan) and wild type HC (4/28; 3% from Mauritania and Oman, 6% from Sudan). Thus, wild type and double mutants of the His (372)–Cys (428) catalytic dyad were equally distributed in the three countries. Similar results were obtained for the His (282)–Cys (305) putative catalytic dyad, with single and double mutants H282**K** and C305**R/T.**

### *Plasmodium vivax* CSP genotyping

Genotyping was successfully performed for *Pvcsp*. The phenotype VK210 was present in all samples with either VK210A (GDRADGQPA) or VK210B (GDRAAGQPA).

## Discussion

This study is the first to look for a link between *Plasmodium* apoptosis and drug resistance through comparison of polymorphisms in metacaspase 1 gene of *P. vivax* and *Pvmdr1* as a marker of resistance. *Plasmodium vivax* isolates were collected in three endemic and non-endemic countries: Mauritania (West Africa), Sudan (northeastern Africa) and Oman (southwestern Asia).

The Nomenclature committee on cell death has defined cell death as “accidental” or “regulated” [37] to avoid misuse of the terms apoptosis, necrosis or autophagy. Accidental cell death is caused by severe physical, chemical or mechanical insults. Regulated cell death may present both apoptotic and necrotic traits that and can be modulated by drugs or genetic intervention [22]. Apoptosis is defined as a caspase dependant variant of regulated cell death, triggered by intrinsic or extrinsic events. Apoptosis is highly controlled, thus reversible, and has been clearly associated with mechanisms of drug resistance in cancer cells. Metacaspases are orthologs of caspases recently identified in protozoan parasites, including the two human malaria parasites, *P. falciparum* and *P. vivax*. The *P. falciparum* metacaspase 1 protein (PfMCA1) is the most studied and its involvement in apoptosis has been demonstrated previously [38]. It was demonstrated that cell death induced by PfMCA1 is aspartate-dependent protease activity. Interestingly, in vitro chloroquino-resistant clone of *P. falciparum* was reported to lack the aspartates dependent proteolytic activity [34].

Therefore, in this study, the polymorphisms found within the PvMCA1-cd gene was compared to the polymorphisms of *pvmdr*1 gene associated with *P. vivax* drug resistance. Samples were collected from three different countries with a low suspected rate of documented treatment failures, to be able to detect mutations arising before fixation.

Analysis of *pvmdr1* sequences has shown SNPs at three codons 958, 976 and 1076 as previously described [6, 12, 15, 17, 19]. The substitution in the codon T958**M** is known as an allelic variant, of which T958 wild-type was identified in Ecuador and 958M is thought being characteristic of Asia and Africa [12], while it has also been found in samples from Brazil [19]., The allelic form 958M was identified in samples from Africa and south western Asia tested here. Few isolates showed the single mutation Y976**F** (4/30), in agreement with the fact that the single 976**F** mutant is not very common worldwide [17, 19]. In contrast, many studies have reported a high prevalence of the Y976**L** and/or F1076**L** mutants associated with treatment failure [15, 18]. In Mauritania, mutations at codons Y976**F** and F1076**L** in the *pvmdr1* gene have been reported but no study has established a relationship between these mutations and clinical responses of *P. vivax* to chloroquine [6].

A high prevalence of double and triple mutants was observed in samples from Mauritania, Sudan, and Oman. Combined analysis of the three codons showed only one isolate from Mauritania with the wild-type MYF. Single MY**L** mutants (27%) were present in the 3 countries, while double M**FL** mutants (10%) were only identified in *P. vivax* isolates from Sudan. These results are in agreement with studies reporting the wildtype MYF in samples from Nepal, Ecuador, and Sri Lanka, the single MY**L** mutants in samples from Sudan, Nepal, Sri Lanka, Pakistan, Amazonas and Brazil. The double M**FL** mutants were mainly reported in Sri Lanka. Furthermore, three new nonsynonymous mutations were also identified (Y976**V** and F1076**I**/F1076**T**), and the triple **YVT** mutants were the most prevalent (53.3%) and distributed in the three countries while the triple **YVI** mutants (3%) were only found in Mauritania. These unexpected mutations were not described previously and their possible link with drug resistance is unknown.

The results obtained from PvMCA1-cd sequences were surprising since only few *P. vivax* isolates (14%) showed the conserved histidine and cysteine residues at position 372 and 428, while sequence analysis of reference strain *P. vivax* Sal-1 and published strains from Mauritania, Brazil, India and North-Korea showed a conserved catalytic dyad. Interestingly, a second potential putative site of histidine and cysteine residues was found at position 282 and 305, in which histidine and cysteine residues were also conserved in reference strains. The seven nonsynonymous mutations identified in both sites, and found in the three endemic countries, suggested that the catalytic domain is variable, in agreement with a study reporting a polymorphism in the full-length of *P. vivax* metacaspase1 [39].

**Fig. 2:**
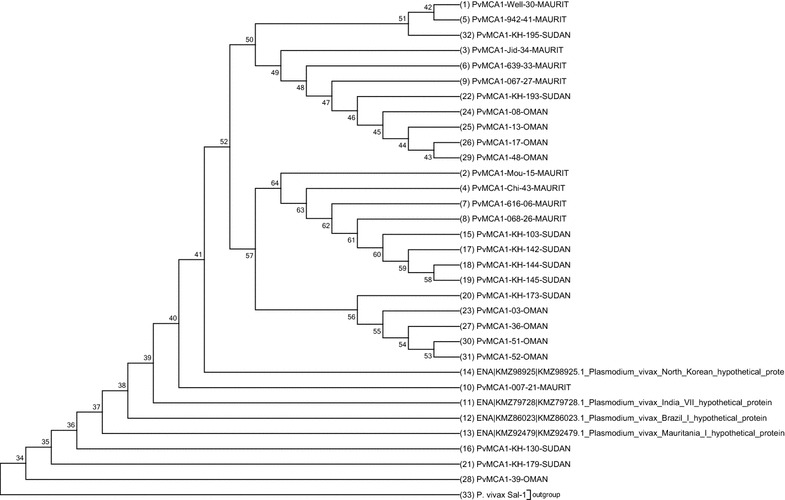
Molecular phylogenetic analysis by maximum likelihood method rooted on *P. vivax* Sal-1. The evolutionary history was inferred by using the Maximum Likelihood method based on the Tamura-Nei model. The tree with the highest log likelihood (−1783.1245) is shown. Initial tree(s) for the heuristic search were obtained automatically by applying Neighbor-Join and BioNJ algorithms to a matrix of pairwise distances estimated using the Maximum Composite Likelihood (MCL) approach, and then selecting the topology with superior log likelihood value. The analysis involved 33 nucleotide sequences. Codon positions included were 1st + 2nd + 3rd + Noncoding. All positions containing gaps and missing data were eliminated. There were a total of 426 positions in the final dataset. Evolutionary analyses were conducted in MEGA7

Phylogenetic tree rooted on *P. vivax* Sal-1 was built using PvMCA1-cd sequences, which showed clearly three new taxa, suggesting a divergence occurred between the five *P. vivax* references strains (Salvador, Mauritania, Brazil, India, North-Korea) and the isolates from Mauritania, Sudan and Oman (Fig. 2). But, the historical occurrence of these polymorphisms in these areas remains unknown.

To identify the reviviscence phenotype of this *P. vivax* parasite population circulating in the three countries, genotyping was done using the *Pvcsp* (circumsporozoite protein). The circumsporozoite protein gene of *P. vivax* comprises a central repetitive domain flanked by two conserved domains [40]. The repetitive domain is composed of a 27 bp element repeated a variable number of times. The VK 210 type (type I: GDRADGQPA) and the VK 247 type (type II: ANGAGNQPG) are the most useful marker for *pvcsp* genotyping. All our samples displayed the VK210 type with different allelic associated with sequences repeated from 9 to 17 times. Surprisingly, all the isolates showed the VK210 pure genotype, demonstrating a temperate phenotype. Long latency *P. vivax* are known to be more widespread than generally thought, including in North Africa, the Horn of Africa, the Middle East and central India [41]. Determination of this periodicity is of utmost importance to adapt the duration of the primaquine regimen for radical cure.

Isolates presenting both quadruple (PvMCA1-cd) and triple (*Pvmdr1*) mutations were observed in Mauritania (5/9), in Sudan (3/9) and in Oman (1/9), leading to the idea that there is no clear difference in the haplotypes from the three areas with the present sample size. While the number of samples is too low to draw conclusion, there is a trend for more mutation in Mauritania.

## Conclusion

The aim of this study was to look for a potential link between single nucleotide polymorphisms in the PvMCA1-cd and *Pvmdr1* genes, in order to test the hypothesis of a role for apoptosis or regulated cell death in *P. vivax* drug resistance. Triple or quadruple mutants for metacaspase genes and double or triple mutants for *Pvmdr1* were observed in most samples. There was no difference in the frequency of mutations between PvMCA1-cd and *Pvmdr1* (P > 0.2). Most of the triple and quadruple mutants for H/C catalytic dyad were also double or triple mutants for *Pvmdr1* (18/24, 75%). Among the four isolates with the conserved histidine and cysteine at both catalytic sites, three were single mutants in the *pvmdr1*gene MY**L,** and one was double mutant M**FL**. These results showed that mutations in metacaspase gene appeared to evolve with a similar frequency than *Pvmdr*1 gene.

The study was based on retrospective analysis of blood samples from patients and no information on treatment and follow-up were available. Thus, the exact link between apoptosis and drug resistance was not discernible. However, a trend for both mutants to be associated is suspected, which supports the interest for further investigations to explore the effects of mutations in the histidine and cysteine catalytic dyad of the PvMCA1 protein on *P. vivax* drug resistance.
